# Optical thin film coated organic nonlinear crystal for efficient terahertz wave generation

**DOI:** 10.1038/s41598-022-17893-7

**Published:** 2022-09-05

**Authors:** Hirohisa Uchida, Tetsuya Kawauchi, Gemma Otake, Chisa Koyama, Kei Takeya, Saroj R. Tripathi

**Affiliations:** 1 ARKRAY Inc., Kamigyo-ku, Kyoto, 602-0008 Japan; 2 Department of Electronics, Nagoya University, Nagoya, Aichi 464-8603 Japan; 3 Department of Mechanical Engineering, Shizuoka University, 3-5-1 Johoku, Hamamatsu, Shizuoka 432-8561 Japan; 4 Institute for Molecular Science (IMS), 38 Nishigonaka, Myodaiji, Okazaki 444-8585 Japan; 5 Graduate School of Science and Technology, Shizuoka University, 3-5-1 Johoku, Hamamatsu, Shizuoka 432-8561 Japan

**Keywords:** Engineering, Materials science, Optics and photonics, Physics

## Abstract

In the process of terahertz (THz) wave generation via optical rectification of infrared femtosecond pulses in a non-linear optical crystal, the power of terahertz wave is directly proportional to the square of the optical pump power. Therefore, high power terahertz wave can be generated using a high power femtosecond laser provided that the crystal has both high laser induced damage threshold and optical non-linear coefficient. However, a significant amount of pump power is lost in this process due to the Fresnel’s reflection at the air-crystal boundary. In this paper, we numerically and experimentally demonstrate that the coat of optical thin film called Cytop on the 4-N, N-dimethylamino-4’-N’-methyl-stilbazolium tosylate (DAST) crystal effectively reduces the reflection loss of pump power, thereby increasing the THz wave emission efficiency of the DAST crystal. We found that the average power of THz wave emitted by the thin film coated crystal is about 28% higher than the THz power emitted by the uncoated crystal when an equal amount of laser power is used. The thin film coated DAST crystals can be used not only in terahertz measurement systems but also in optical devices such as modulators and waveguides.

## Introduction

The applications of terahertz (THz) wave have been expanding at an enormous speed in various fields such as biomedical engineering, non-destructive testing, high speed communication and ultrafast spectroscopy^1–4^. In these applications, it is important to use a highly efficient terahertz wave source to improve the performance of the measurement system. Various sources such as photoconductive antenna^5^, non-linear optical crystals^6^, quantum cascade lasers^7^ have been developed to emit terahertz wave. Moreover, other sources based on laser plasma interaction^8^, terahertz spintronics^9^ and terahertz superconductors^10^ are also being investigated. Among them, nonlinear optical crystals are very promising for broadband THz wave generation via optical rectification^11^, difference frequency generation^12^ and optical parametric processes^13^. Particularly, optical rectification of femtosecond (fs) laser pulses in non-linear optical crystals has attracted special attention owing to its ability to emit broadband and high-power THz wave.

Both inorganic and organic crystals have been used for THz wave generation via optical rectification. For example, Lithium Niobate is a well-known inorganic non-linear optical crystal that can produce high power THz waves^14,15^. Similarly, Zinc Telluride^16–18^, Gallium Phosphide^19^ and Gallium Arsenide^20^ have also been widely used to emit broadband THz waves. However, these crystals have limitations such as low conversion efficiency due to their moderate nonlinear coefficients and increased system complexity such as in the case of THz wave generation using Lithium Niobate crystal via tilted front of laser pulses^21^. Compared with these inorganic crystals, organic crystals such as DAST, HMQ-TMS, DSTMS, OH1, and BNA have proven to be an excellent THz sources because of their characteristics such as high optical to THz conversion efficiency due to high nonlinear optical coefficient, low terahertz wave absorption, and relatively simple measurement system due to collinear phase matching geometry^22–26^. Among these organic crystals, DAST is one of the widely used organic crystals for THz wave generation because of its high optical non-linear coefficient (d_11_ = 290 ± 55 pm/V at λ = 1.5 μm), low optical as well as terahertz wave absorption and high laser induced damaged threshold^27–29^. Moreover, this crystal can be pumped by well-established and widely available telecom fiber laser with a wavelength of 1.5 μm^30,31^.

Generation of THz wave from non-linear crystals via optical rectification of fs laser pulses is based on difference frequency mixing of all frequencies within the bandwidth of a fs laser pulse. In this second order non-linear optical process, the power of THz wave is directly proportional to the square of the power of femtosecond pump laser^32,33^. Therefore, high intensity THz wave can be generated using a non-linear optical crystal which has high laser induced damage threshold, high optical non-linear coefficient and low THz wave absorption. However, THz emission efficiency of these crystals is limited by reflection loss of pump power. When a pump laser is incident on a crystal surface, a significant percentage of pump power is reflected from air-crystal interface given by Fresnel’s equation as ${\left(\frac{n-1}{n+1}\right)}^{2}$, where *n* is the refractive index of a crystal at the excitation wavelength. However, this reflection loss can be reduced by using an anti-reflection coat on the surface of the crystal with an appropriate thickness^34^.

Anti-reflection coating is primarily utilized to suppress Fresnel’s reflection losses when light propagates from one medium to another medium and such coat can be made either by depositing thin film on the surface of the crystal^35,36^ or by using graded refractive index coating with sub-wavelength structures such as moth eye structures^37,38^. In this paper, we report a dielectric film type antireflection coating called Cytop, its coating process and its role as an anti-reflection coating. We numerically and experimentally demonstrate that such coat help reduce the significant amount of power loss due to reflection and ultimately enhance the terahertz wave emission efficiency of non-linear optical crystals.

## Anti-reflection coating

### Film thickness calculation

**Figure 1:**
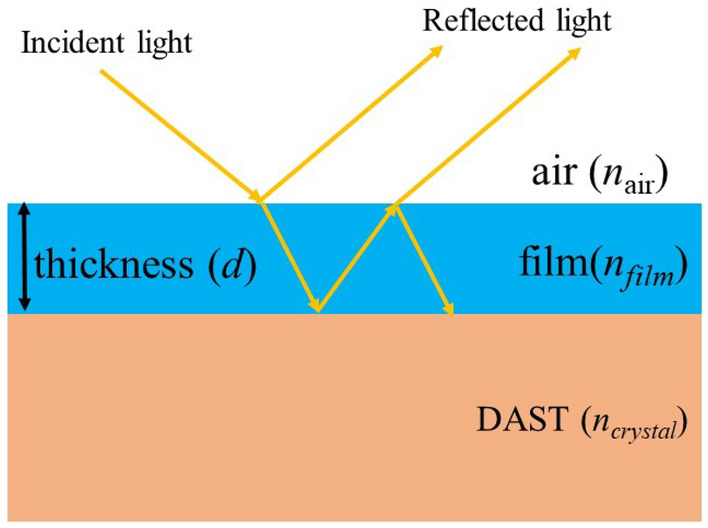
Schematic diagram of a thin film antireflection coating on the DAST crystal.

The anti-reflection coating on a non-linear optical crystal can be realized by a single layer dielectric thin film. When a film is coated on a crystal, the thin film creates two interfaces: air-film and film-crystal and these interfaces produce two reflected waves as shown in Fig. 1. When these two reflected waves have same intensity with their phase difference of π, the total energy of reflected waves becomes zero due to the destructive interference thus enhancing the transmittance. In order to realize this condition, the refractive index of film should be lower than the refractive index of the crystal in the wavelength of interest, written as *n*_*film*_ < *n*_*crystal*_. Moreover, the optical thickness of the thin film must be an odd multiple of quarter wavelength (*n*_*film*_*.d* = *λ*/4), where *λ* is the wavelength of the incident laser.

At the normal incidence of light, the reflectance is written as^39^

1 $${R=\left(\frac{{n}_{air}{n}_{crystal}-{n}_{film}^{2}}{{n}_{air}{n}_{crystal}+{n}_{film}^{2}}\right)}^{2}$$

where *n*_*air*_ is the refractive index of the air, *n*_*crystal*_ is the refractive index of the crystal and *n*_*film*_ is the refractive index of the thin film. In order to make the value of *R* = 0, the numerator of the right term should be written as

2 $${n}_{air}{n}_{crystal}={n}_{film}^{2}$$

Therefore refractive index of the film (*n*_*film*_) can be obtained as

3 $${n}_{film}=\sqrt{{n}_{air}{n}_{crystal}}$$

In the case of the DAST crystal (*n*_*crystal*_ = 2.13 at λ = 1560 nm), the refractive index of the film (*n*_*film*_) is computed as √2.13 = 1.45. In this study, we chose a polymer called Cytop as an antireflection coat as its refractive index (*n*_*film*_ = 1.33 at λ = 1560 nm) is close to the required refractive index obtained using Eq. 3. Finally, the required optical thickness of the film (*d*) is computed as *λ*/(4.*n*_*film*_) ≈ 293 nm.

### Anti-reflection film coating process

**Figure 2:**
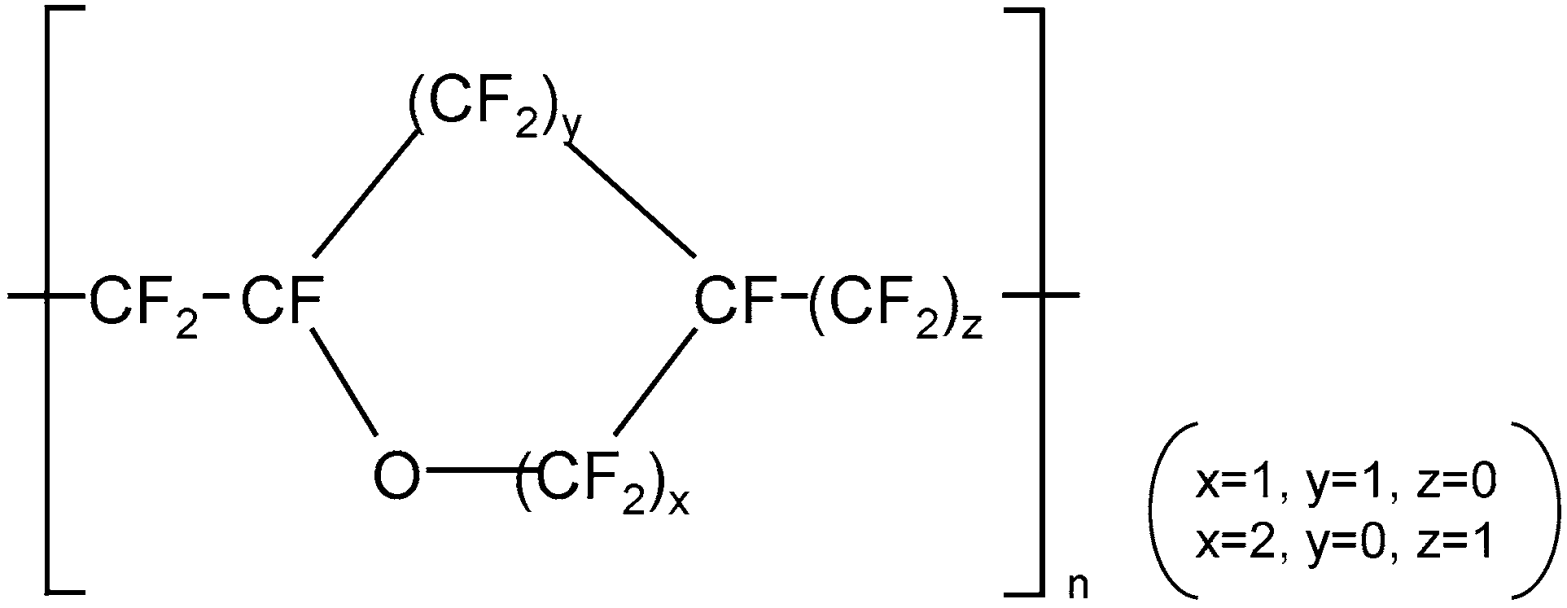
Chemical structure of Cytop.

The structural formula of Cytop™ (manufactured by AGC chemical Inc.) is shown in Fig. 2. Cytop is an amorphous fluoropolymer that dissolves in fluorine-based solvents, and it can be used as a thin film coating with thicknesses of few hundreds of nanometers. Various coating methods such as spray coating, spin coating, dip coating and die coating can be used depending upon the material and its surface roughness. Cytop has a refractive index of 1.33 in infrared region and 1.43 in terahertz frequency region^40^. Moreover, it has low absorption coefficient in both infrared and terahertz frequency region making it suitable material for anti-reflection coating for non-linear crystal for terahertz wave generation (for other properties of Cytop, please refer Supplementary information S1).

**Figure 3:**
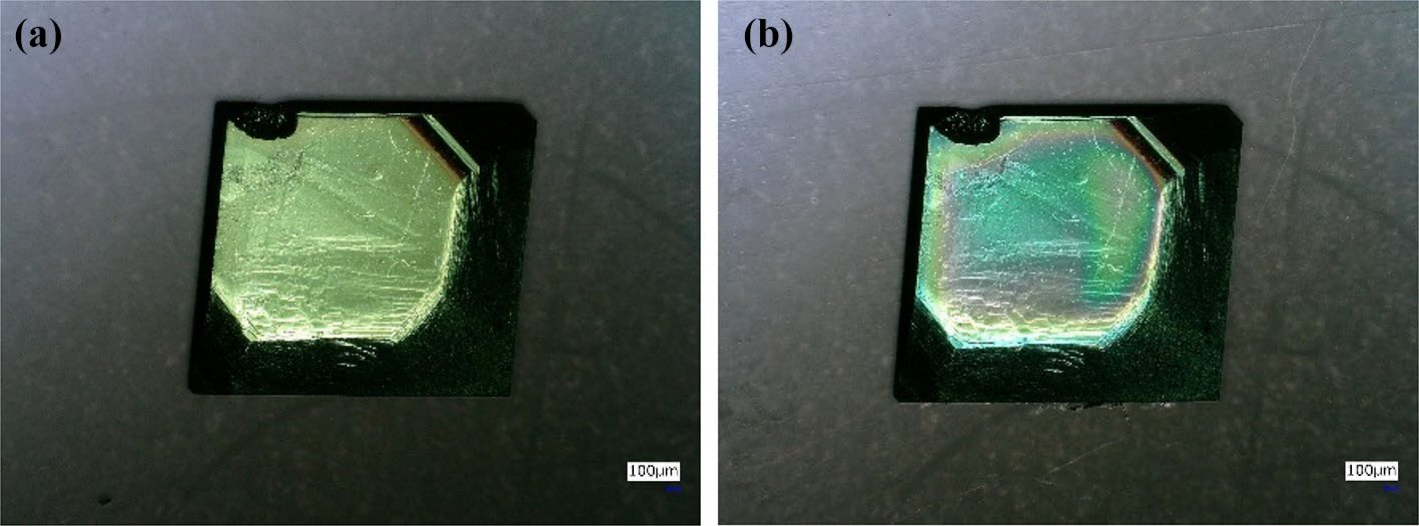
(**a**) Uncoated DAST crystal, (**b**) Cytop film coated DAST crystal.

In this experiment, dip coating method has been implemented to coat the DAST crystal with a Cytop as an anti-reflection coating. In the first step, the Cytop solution (CTL-109AC, manufacturer: AGC chemical Inc.) was dissolved in a solvent (CT-SOLV100E, manufacturer: AGC chemical Inc.) and Cytop solutions of different concentrations (0%, 1%, 3%, 5%, 7% and 9%) were prepared. Next, the DAST crystals were immersed in the Cytop solution and these crystals were taken out of the solution at a pulling rate of 1 mm/sec. These crystals were dried naturally at room temperature for 5 min and then dried in an over for 1 h at 100 °C. Figure 3 shows the uncoated and Cytop film coated DAST crystals. Further detail of the coating process is given in “Methods” section.

In the next step, we measured the thickness of the Cytop coating using an ellipsometer (UVISEL2, Manufacturer: Horiba Ltd.) and we studied the coating thickness dependency on the concentration of Cytop solution as shown in Table 1. We also calculated the coefficient of variation (CV) (calculated as: CV = standard deviation/mean × 100%) to examine the uniformity of optical film thickness. The table shows that the coefficient of variation decreases with the increase in coat thickness up to the solution concentration of 7%. Beyond that the CV increases as it is difficult to control the thickness of the thin film in dip coating method. As discussed in the previous section, the required optical thickness of the Cytop coating is 293 nm, we used the DAST crystal coated with Cytop solution with a concentration of 5%. The CV is 2.4% for the crystal with a coat thickness of 288 nm, showing a good uniformity of the film thickness.

**Table 1:** Variation of solution concentration and coating thickness.

Solution concentration (%)	Coating thickness (nm)	Standard deviation (σ)	Coefficient of variation (%)
1	6.3	4.21	66.8
3	56.3	5.6	9.9
5	288.6	6.8	2.4
7	852.6	13.7	1.6
9	1363	78	5.7

Since the DAST crystal is hygroscopic in nature, special attention needs to be paid while storing the crystals. Therefore, we studied the effect of humidity on the quality of coated and uncoated crystals by placing them in a controlled humidity chamber (relativity humidity = 80%, temperature = 30 °C) for 7 days. The crystal hydrates are formed on the surface of the uncoated crystal whereas no significant change was observed on the surface of the coated crystal. This result indicates that the Cytop film coated DAST crystal is resistant to humidity and moisture in comparison to the uncoated DAST crystal.

## Evaluation of DAST crystal

In order to evaluate the performance of the Cytop thin film as an anti-reflection coating, we first numerically investigated the reflection and transmittance properties of the laser through the crystal with and without the AR coating.

### Case I: without anti-reflection coating

When a laser beam is incident on a crystal, the reflection of laser beam from the air-crystal boundary is computed using Fresnel’s equation as^39^

4 $${R=\left(\frac{{n}_{air}-{n}_{crystal}}{{n}_{air}+{n}_{crystal}}\right)}^{2}$$

Here, *n*_*air*_ = 1, and *n*_*crystal*_ = 2.13 at *λ* = 1560 nm for the *a*-axis of the DAST crystal, which gives total reflectance = 13.03%. Now, the transmittance of the crystal is computed as

5 $$T={\left(1-R\right)}^{2}\times \mathrm{exp}\left(-\alpha d\right)$$

Here, *α* is the absorption coefficient of DAST crystal (0.7 cm^−1^ at *λ* = 1560 nm) and *d* is the thickness of the crystal (0.5 mm). The total transmittance of the uncoated DAST crystal is calculated as 73.03%.

### Case II: with anti-reflection coating

When a crystal is coated with the Cytop thin film on both sides with the refractive index of 1.33 at λ = 1560 nm, the reflectance is computed as

6 $${R}^{^{\prime}}{=\left(\frac{{n}_{air}-{n}_{opt}}{{n}_{air}+{n}_{opt}}\right)}^{2}-\frac{4{n}_{air}{n}_{opt}\left({{n}_{AR}}^{2}-{{n}_{air}}^{2}\right)\left({{n}_{opt}}^{2}-{{n}_{AR}}^{2}\right)}{{\left({n}_{air+}{n}_{opt}\right)}^{2}{\left({{n}_{AR}}^{2}+{{n}_{air}n}_{opt}\right)}^{2}}$$

where the reflectance is computed as 0.85%. Finally, the transmittance is computed as 94.91% using the equation written below

7 $${T}^{^{\prime}}={\left(1-{R}^{^{\prime}}\right)}^{2}\times \mathrm{exp}\left(-\alpha d\right)$$

From the comparison, we found that the transmittance of the crystal is increased to 94.91% with the AR coating, whereas the transmittance is only around 73.03% without the AR coating. This clearly demonstrates that the AR coating is effective in reducing the reflection loss of pump beam.

In order to experimentally evaluate the transmittance of the laser light from the DAST crystal, we constructed a measurement setup using a femtosecond laser with wavelength of 1560 nm and average power of 80 mW (the detail of the experimental setup is given in supplementary information S1). The laser beam was focused to the crystal using a lens with focal length of 50.8 mm and the laser light transmitted through the crystal is detected using optical power meter (FieldMax II, Coherent Inc.). We measured the transmittance of both crystals (*d* = 0.5 mm) with and without anti-reflection coating. The transmittance values of the crystal with and without the AR coating are obtained as 93.4% and 74.3% respectively. This indicates that the transmittance increased approximately by 26% when the AR coated DAST crystal is used. These experimental results show good consistency with the numerically computed values. Since the damage threshold is an important parameter while evaluating the performance of the DAST crystal, we have previously studied the laser induced damage threshold at λ = 1560 nm for DAST crystal coated with Cytop thin film. AR-coated DAST crystals were shown to withstand laser irradiation with a power density of 3.6 GW/cm^2^ for 720 minutes^36^. Therefore, laser induced damage on the crystal was not observed in this experiment. Here it is important to note that the AR coated DAST crystal is not only useful in THz generation but also in other applications employing laser such as high-speed optical modulators and field detectors.

## Power measurement

**Figure 4:**
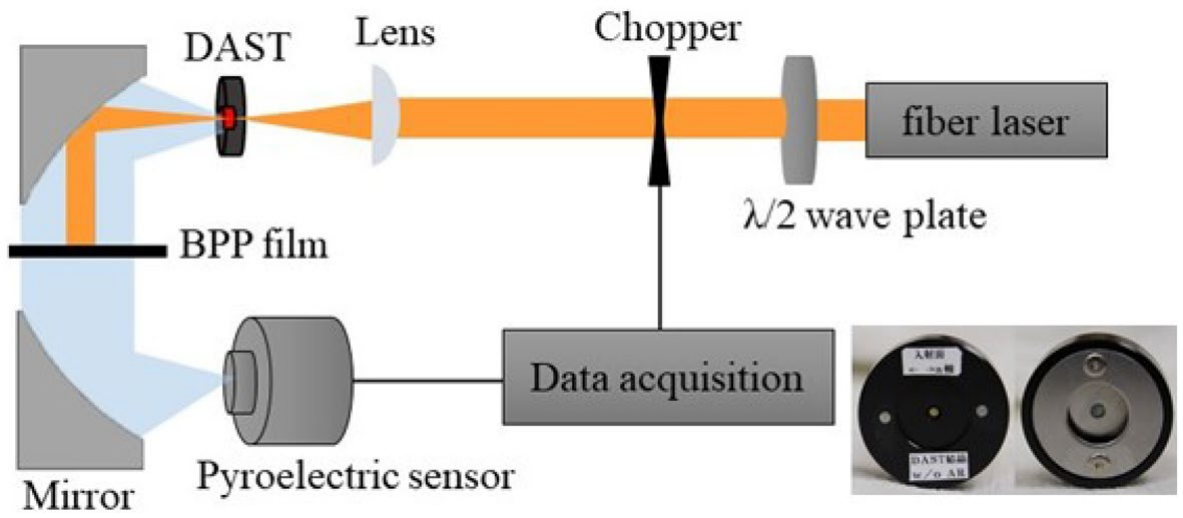
Schematic diagram of the optical setup to measure the power of THz wave. Inset shows the DAST crystals mounted on the crystal holders.

Next, we measured the terahertz wave emitted by both crystals and compared the average power of terahertz waves emitted by these crystals. The experimental setup is shown in Fig. 4, where we used a femtosecond fiber laser (KPhotonics LLC.) with wavelength of 1560 nm, pulse width of less than 55 fs, pulse repetition rate of 50 MHz and average power of 80 mW. The femtosecond laser was focused on the DAST crystal to a spot diameter of 60 μm using a lens with the focal length of 50.8 mm. The DAST crystal is fixed in a holder as shown in the inset of Fig. 4. We used *λ*/2 wave plate to align the polarization of laser light to the *a*-axis of the DAST crystal. The emitted terahertz wave was first collimated by off-axis parabolic mirror and focused to the calibrated pyro-electric detector (Gentec Inc.). The laser light transmitted through the DAST crystal was blocked by black polypropylene sheet, which has a transmittance of 0% and 70% for laser and THz wave respectively (See supplementary information S1). The pump beam was modulated by a chopper with a frequency of 5 Hz.

**Figure 5:**
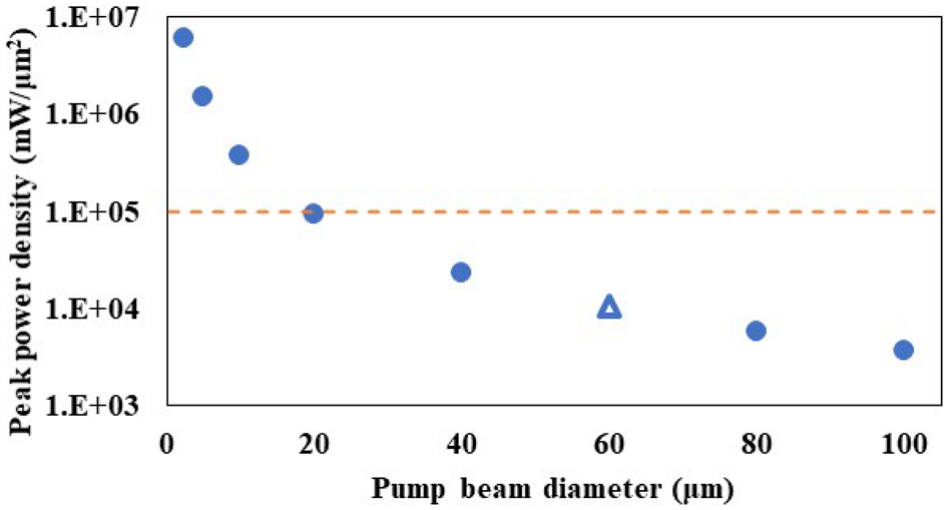
Relationship between pump beam diameter and peak power density. The triangle shows an experimental value in this study. Power density of 10^5^ mW/μm^2^ is shown by a dotted line for a reference.

Femtosecond lasers are often used in high-power terahertz wave generation via a non-linear optical process. Therefore, the peak power density of the laser at focal point of the lens is significantly high, inducing non-linear scattering. Such scattering effects are considered to occur at a peak power density of 10^5^ mW/μm^2^ or more^41^. Here, we studied the relationship between pump beam diameter and peak power density in our experiment as shown in Fig. 5. This indicates that the beam diameter at which the power density becomes greater than 10^5^ mW/μm^2^ is 19.2 μm when a laser with a pulse width of 55 fs, pulse repetition rate of 50 MHz, and average power of 80 mW is used. Therefore, the effect of nonlinear scattering is considered to be almost negligible since the beam is focused at 60 μm in this experiment. Increasing power density is usual practice in terahertz waves generation process using non-linear optical crystal. Therefore, this result shows that the relationship between nonlinear scattering effect and the peak power density should be carefully considered when the thin-film coated nonlinear optical crystal is pumped with high power laser for terahertz wave generation.

**Figure 6:**
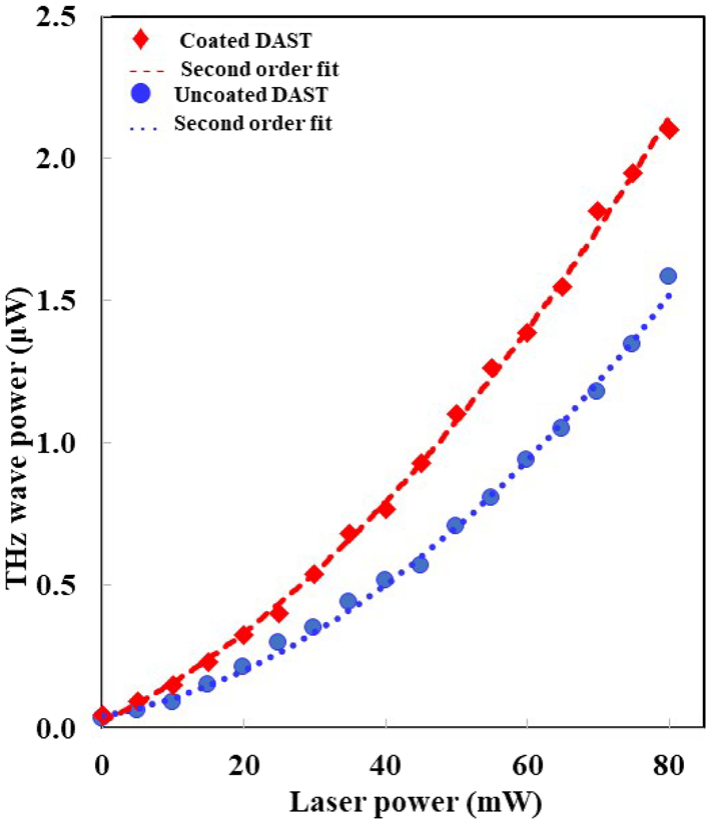
Average power of terahertz wave emitted by antireflection film coated and uncoated DAST crystals.

Figure 6 shows the dependency of average terahertz power emitted by both DAST crystals, with and without AR coating, on the incident pump power. The power of the laser is varied using a neutral density filter from 0 to 80 mW. Since the power of the THz wave is directly proportional to the square of the power of the pump laser, a second-degree polynomial equation was used to fit the data as shown in Fig. 6. Here, it is observed that the ratio of THz power emitted by coated DAST crystal to that of uncoated crystal remains approximately constant within the measurement range. The maximum power is about 2.08 μW and 1.62 μW respectively for coated and uncoated DAST crystal when pumped with laser power of 80 mW. This shows that the average power of THz wave emitted by AR coated crystal is 28% higher than the THz power emitted by crystal without the AR coating when an equal amount of laser power is used. Since the laser transmittance was increased by approximately 26% when the coated DAST crystal was used, 28% increase on THz power obtained from this experiment is reasonable. Moreover, we investigated the optical-to-terahertz power conversion efficiency (calculated as: efficiency = average terahertz power / average laser power × 100%) when the crystals were pumped with the maximum laser input power. We obtained the AR coated crystal has a conversion efficiency of 0.0026% whereas the uncoated crystal has an efficiency of 0.0020%. This also shows the importance of a thin-film coating on a non-linear optical crystal for efficient terahertz wave generation. Overall, it is evident that the emission efficiency of DAST crystal can be improved by using the AR coating on it. The THz power emitted by the DAST crystal without AR coating is consistent with our previously reported result^42^. Comparing these results, more than 20 μW is expected from the AR coated crystal when pumped with a femtosecond laser with a power of about 280 mW.

**Figure 7:**
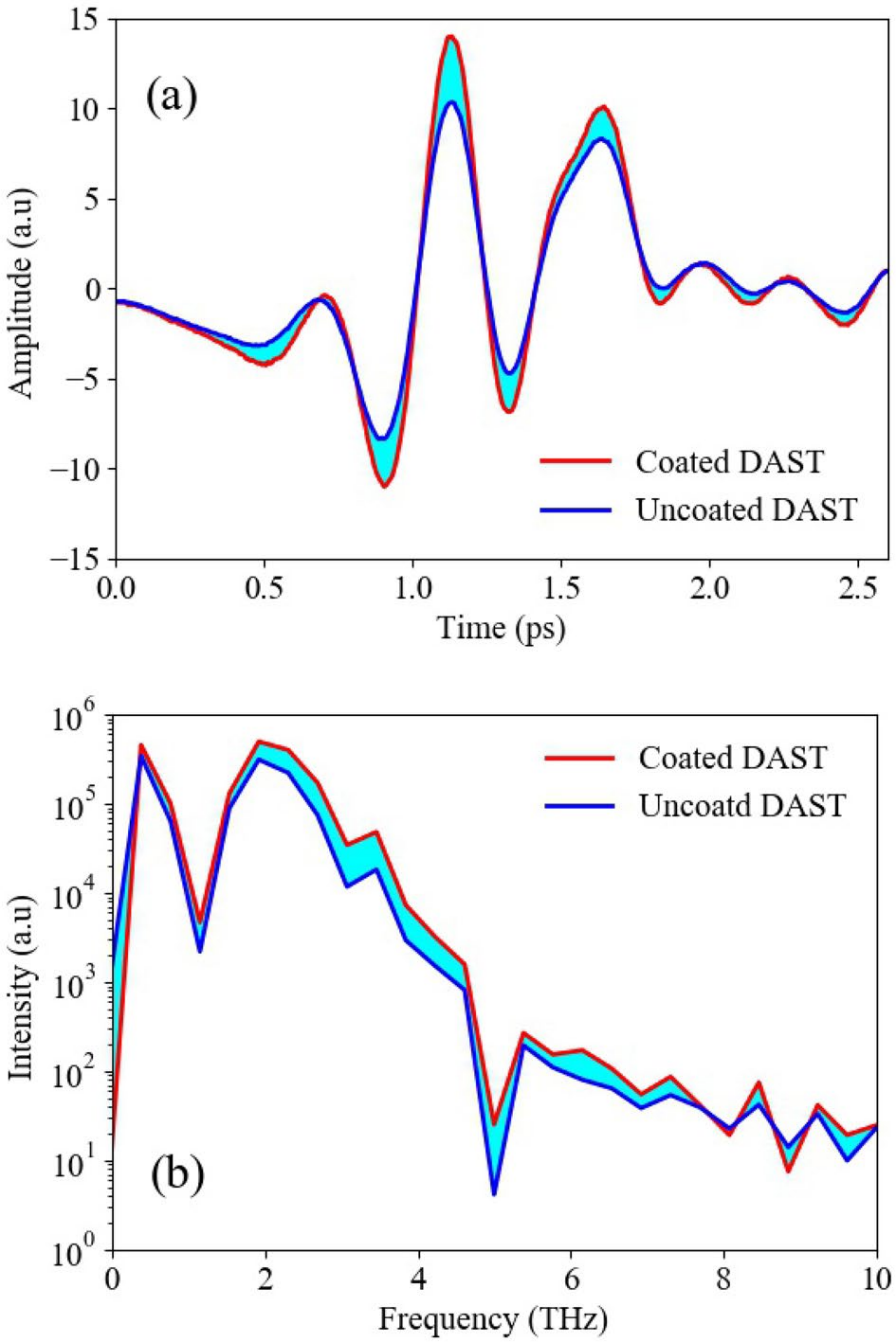
(**a**) THz time domain pulse emitted by AR coated and uncoated DAST crystals and (**b**) their respective intensity spectra. The shaded area shows the difference in terahertz intensity emitted by the two crystals.

Next, we developed standard terahertz time domain spectrometer in order to compare the time domain electric field and intensity spectrum obtained by both crystals. The terahertz waves emitted by both AR coated and uncoated DAST crystal are coherently detected by photoconductive antenna (the detail of measurement setup is given in “Methods” section). The time domain electric field of the THz pulse is recorded by changing the relative time between pump and probe pulse using mechanical delay stage. Figure 7a shows the time domain THz pulses emitted by both crystals. Here, the peak-to-peak amplitude of THz pulse emitted by coated and uncoated crystal are 25.0 and 18.7 respectively, indicating that the THz pulse amplitude is enhanced by the factor of 1.3. We also obtained the intensity spectra of these THz electric pulses using fast Fourier transformation as shown in Fig. 7b. The frequency range extends from 0.2 THz to about 8 THz and it is evident that the THz intensity is increased over the entire frequency range indicating that the enhancement of terahertz wave emission efficiency is independent of the frequency. Here it is worthwhile to note that the dips around 1.1 THz and 5.2 THz in the intensity spectra are due to the transverse optical phonons in the DAST crystal originating from the ionic bonds^28,43^.

## Conclusion

AR coating is a well-established method to avoid the surface reflection, but its potential has never been exploited to enhance the efficiency of non-linear optical crystal for THz wave emission. In this study, we have presented a method to enhance the efficiency of terahertz wave generation by using an anti-reflection coat on DAST crystal. We numerically and experimentally demonstrated that the transmittance of the laser through the AR coated crystal is about 1.26 times higher than with the uncoated crystal, indicating that the Fresnel’s reflection is significantly reduced using anti-reflection coating. In the next step, we measured the terahertz wave generated using the crystals with and without AR coating and we confirmed that the average power emitted by AR coated crystal is approximately 28% higher than that of THz wave emitted using crystal without AR coating when equal amount of laser power is used to excite the DAST crystals. Here, we demonstrated that the Cytop film coated DAST crystal can be used for efficient THz wave generation but such AR coated DAST crystals can also be used in other optical applications such as high-speed optical modulators and electric field detectors in order to improve the efficiency of the system.

## Methods

### Cytop coating process

Dip coating method was used to coat the DAST crystal, where the most important step is to prepare a cytop solution of desired concentration. In our study, 5% of cytop solution was used to obtain the antireflection coating thickness of 288 nm. In order to prepare 5% cytop solution, first 1.029 mL of CTL-109AE (AGC Chemical Inc.) was measured using a micropipette, which was then mixed with 2.5 mL of solvent CT-SOLV100E (AGC Chemical Inc.). This solution was placed in a dip coater. The system was designed in such a way that the sample can be pulled up or down at a desired speed. The Cytop solution was placed so that the entire DAST crystal was immersed in the Cytop solution. Here, an elastomer sheet was attached to the bc-plane of the DAST crystal and dipped into the solution. The pull-down and pull-up rates of the DAST crystal are 3.5 mm/s and 1.0 mm/s, respectively. After dipping, the DAST crystals were allowed to dry naturally for 5 min and removed from the dip coater. Finally, crystals were dried in an oven at 100 °C for an hour.

### Terahertz time domain spectrometer

We developed standard terahertz time domain spectrometer to record the time domain profile of THz waves emitted by coated and uncoated DAST crystal. The laser beam generated by fiber laser (KPhotonics LLC. λ = 1560 nm, pulse width < 60 fs, pulse repetition rate = 50 MHz and average power = 80 mW) was divided into 3:1 using polarization maintaining fiber coupler. The strong beam is used to pump and DAST crystal where the polarization of the laser was aligned to the a-axis of the DAST crystal using a half wave plate. This laser is then focused to the DAST crystal using a lens with a focal length of 50.8 mm. The emitted THz wave is then collimated using off axis parabolic mirror and focused to the photoconductive antenna using another off axis parabolic mirror. The second half of the laser beam travels through the optical delay line and coupled into the photoconductive antenna (Menlo Systems GmbH, TERA 15-RX-FC). The laser beam transmitted through the DAST crystal is blocked using black polypropylene (BPP) film which has a transmittance of 0% at laser wavelength, whereas its transmittance at 2 THz is around 70%. The time domain pulse is recorded by changing the relative time between pump and probe pulse using mechanical delay stage.

## Supplementary Information

Supplementary Information.

## Data Availability

The datasets used and/or analyzed during the current study are available from the corresponding author on reasonable request.
