# Supplementary material for: Optical thin film coated organic nonlinear crystal for efficient terahertz wave generation
Source: Sci Rep. 2022 Sep 5;12:15082. doi: 10.1038/s41598-022-17893-7 (PMC9445094; doi:10.1038/s41598-022-17893-7)
Supplement: Supplementary file 1 — Supplementary Information. [file 41598_2022_17893_MOESM1_ESM.docx]

Supporting Information

Optical thin film coated organic nonlinear crystal for efficient terahertz wave generation

Hirohisa Uchida,^1,2,6*^ Tetsuya Kawauchi,^3,6^ Gemma Otake,^3^ Chisa Koyama,^1^ Kei Takeya,^4^ and Saroj R. Tripathi^3,5,**^

^1^ARKRAY Inc., Kamigyo-ku, Kyoto 602-0008, Japan

^2^Department of Electronics, Nagoya University, Nagoya, Aichi 464-8603, Japan

^3^Department of Mechanical Engineering, Shizuoka University, 3-5-1 Johoku, Hamamatsu, Shizuoka 432-8561, Japan

^4^Institute for Molecular Science (IMS), 38 Nishigonaka, Myodaiji, Okazaki 444-8585, Japan

^5^Graduate School of Science and Technology, Shizuoka University, 3-5-1 Johoku, Hamamatsu, Shizuoka 432-8561, Japan

^6^These authors contributed equally.

* uchidah@arkray.co.jp

**sarojrt@outlook.com

**A: Physical properties of Cytop**

Cytop is an amorphous, low molecular weight, fluoropolymer which can be used as a thin film coating with thickness less than 1 μm.^[S1]^ Table S1 shows the various physical properties of Cytop as described in website of AGC chemical Inc. Here it is important to note that the coefficient of thermal expansion of cytop is about two orders of magnitude larger than SiO_2_, a commonly used material for AR coating. Moreover, the transmittance of cytop at *λ*= 1560 nm is higher that of PMMA with the same thickness. These properties make Cytop an excellent material for antireflection coating for organic crystal.

**Table S1.** The physical properties of Cytop.

| Property | Unit | Material [Cytop] |
| --- | --- | --- |
| Glass transition temperature | °C | 108 |
| Specific gravity | - | 2.03 |
| Water absorption | % | <0.01 |
| Coefficient of thermal expansion | K^-1^ | 7.4 * 10^-5^ |
| Refractive index @ 1560 nm | - | 1.3335 |
| Transmittance @ 1560 nm  (PMMA with a thickness of 200 µm) | % | 95 |

**B: Terahertz Properties of the Cytop**

The refractive index and absorption coefficient of cytop were obtained using standard terahertz time domain spectroscopy in transmission mode.^[S2]^


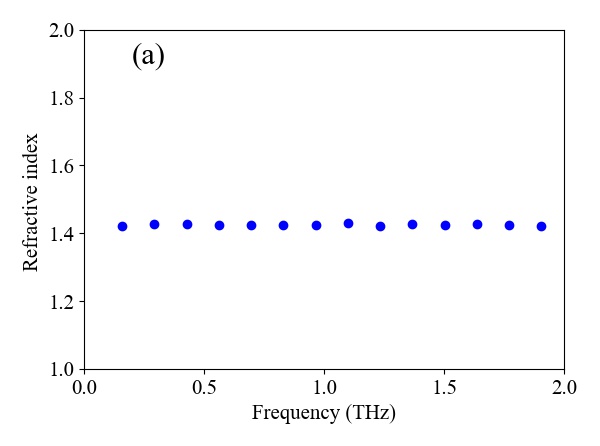


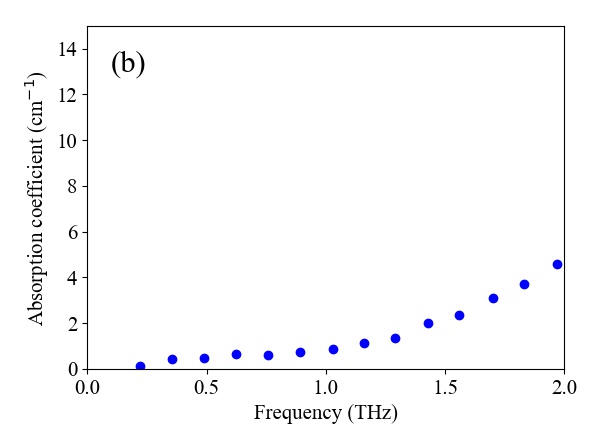


**Figure S1**. Refractive index (a) and absorption coefficient (b) of cytop obtained using terahertz time domain spectrometer

**C: Transmittance of Coated and Uncoated DAST Crystal at 1560 nm**

In order to experimentally evaluate the transmittance of the laser from the DAST crystal, we constructed a measurement setup as shown in Figure S2. Here, the femtosecond laser with wavelength of 1560 nm, with average power of 80 mW was used to irradiate the crystal. The laser beam is focused using lens with focal length of 15 mm and the transmitted laser light is detected using optical power meter (FieldMax II, Coherent Inc.). We measured the transmittance of both crystals (*d* = 0.5 mm) with and without anti-reflection coating. The transmittance values of the crystal with and without the AR coating are obtained as 93.4%. and 74.3 % respectively.


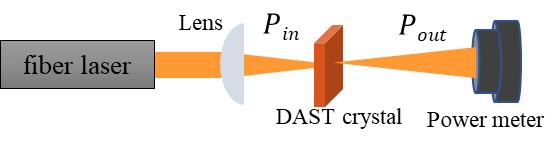


**Figure S2.** Transmission measurement of coated and uncoated DAST crystals at λ= 1560 nm.

**D: Transmittance of black polypropylene in infrared and terahertz region.**

We used black polypropylene to block the laser transmitted through the DAST crsytal. It has a transmittance of 0 % and 70 % around wavelength of 1.5 μm and frequency of 2 THz respectively as shown in the Figures S3 (a) and S3 (b). ^[S3]^


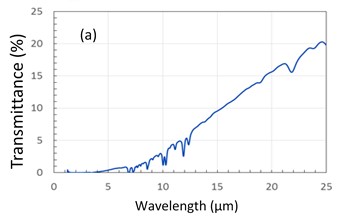


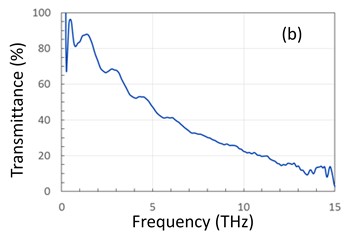


**Figure S3.** (a) Transmittance of the black polypropylene sheet at laser wavelength (λ = 1560 nm) and (b) transmittance of the black polypropylene sheet in terahertz frequency region.

**E: THz time domain spectrometer**

The terahertz time domain spectrometer used to measure the time domain signals emitted by coated and uncoated DAST crystal is shown in Fig. S4.


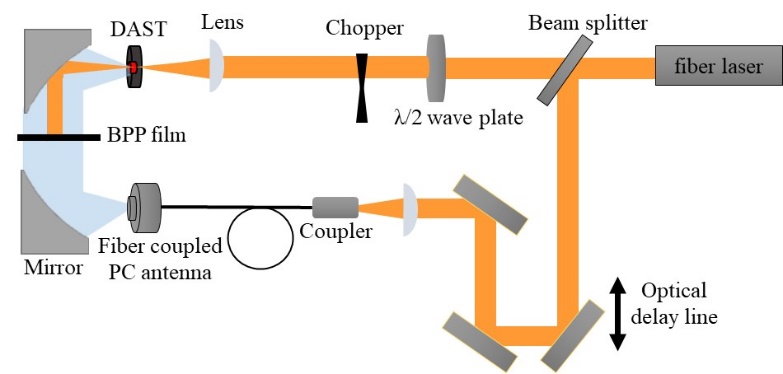


**Figure S4.** (a) Terahertz time domain spectrometer used to measure the time domain signal emitted by both coated and uncoated DAST crystals.

**F: Cytop film coating**

We used dip coating method to coat the thin film on the DAST crsytal. The photograph of the dip coater is shown in Fig. S5.


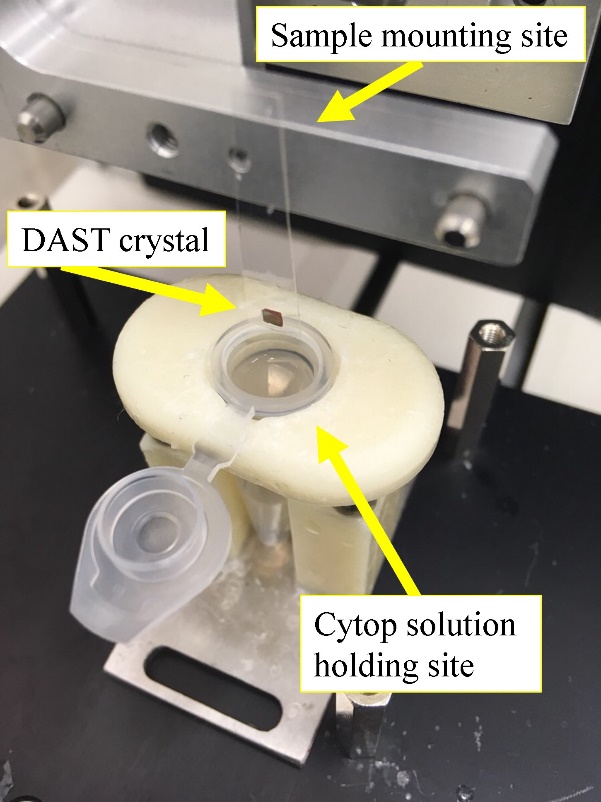


**Figure S5**. Dip coater for Cytop coating.

**References:**

1. M. Yamabe, A challenge to novel fluoropolymers. Macromolecular Symposia **64**, 11-18 (1992)
2. Withayachumnankul W., Naftaly M. Fundamentals of measurement in terahertz time-domain spectroscopy. J. of Inf. Millimeter THz Waves, **35**, 610-637 (2014).
3. http://thz-origin.jp/thz-products.html (Accessed on Feb. 2022)
